# A Novel GTP-Binding Inhibitor, FX2149, Attenuates LRRK2 Toxicity in Parkinson’s Disease Models

**DOI:** 10.1371/journal.pone.0122461

**Published:** 2015-03-27

**Authors:** Tianxia Li, Xinhua He, Joseph M. Thomas, Dejun Yang, Shijun Zhong, Fengtian Xue, Wanli W. Smith

**Affiliations:** 1 Department of Pharmaceutical Sciences, University of Maryland School of Pharmacy, Baltimore, MD, United States of America; 2 Beijing Institute of Pharmacology and Toxicology, Beijing, China; 3 School of Life Science and Biotechnology, Dalian University of Technology, Liaoning, China; Johns Hopkins University, UNITED STATES

## Abstract

Leucine-rich repeat kinase-2 (*LRRK2*), a cytoplasmic protein containing both GTP binding and kinase activities, has emerged as a highly promising drug target for Parkinson’s disease (PD). The majority of PD-linked mutations in LRRK2 dysregulate its GTP binding and kinase activities, which may contribute to neurodegeneration. While most known LRRK2 inhibitors are developed to target the kinase domain, we have recently identified the first LRRK2 GTP binding inhibitor, 68, which not only inhibits LRRK2 GTP binding and kinase activities with high potency *in vitro*, but also reduces neurodegeneration. However, the *in vivo* effects of 68 are low due to its limited brain penetration. To address this problem, we reported herein the design and synthesis of a novel analog of 68, FX2149, aimed at increasing the *in vivo* efficacy. Pharmacological characterization of FX2149 exhibited inhibition of LRRK2 GTP binding activity by ~90% at a concentration of 10 nM using *in vitro* assays. Furthermore, FX2149 protected against mutant LRRK2-induced neurodegeneration in SH-SY5Y cells at 50-200 nM concentrations. Importantly, FX2149 at 10 mg/kg (i.p.) showed significant brain inhibition efficacy equivalent to that of 68 at 20 mg/kg (i.p.), determined by mouse brain LRRK2 GTP binding and phosphorylation assays. Furthermore, FX2149 at 10 mg/kg (i.p.) attenuated lipopolysaccharide (LPS)-induced microglia activation and LRRK2 upregulation in a mouse neuroinflammation model comparable to 68 at 20 mg/kg (i.p.). Our results highlight a novel GTP binding inhibitor with better brain efficacy, which represents a new lead compound for further understanding PD pathogenesis and therapeutic studies.

## Introduction

Parkinson’s disease (PD) is a progressive neurodegenerative disorder, affecting 2% of the population over the age of 60 [1,2]. PD patients display a loss of dopaminergic neurons in the substantia nigra and the presence of Lewy bodies in their brains [1,2]. The current pharmacotherapy for PD patients is limited to symptomatic treatment, which only temporarily reduces motor symptoms but does not prevent neurodegeneration. To date, there are no disease modifying drugs to prevent dopaminergic neuron loss and abnormal protein deposition in the brains. There is a strong demand for neuroprotective therapies to prevent or attenuate dopaminergic neuron degeneration.

Recent genetic studies have identified that mutations in Leucine-rich repeat kinase-2 (*LRRK2*) cause a genetic form of PD and have implications in sporadic PD. LRRK2 is a large cytoplasmic protein (2527 aa) and contains both GTPase and kinase domains [1,2]. Most disease-linked mutations of LRRK2 have been reported to dysregulate its GTP binding and/or kinase activities [2–5]. The most common PD-linked mutation, G2019S, has abnormally elevated kinase activity [1–3,6]. A number of potential LRRK2 kinase inhibitors that target the kinase domain activity have been reported [7–11] and some of them can ameliorate neurodegeneration [8,10]. However, none of these are available in the clinic yet due to poor specificity or low *in vivo* efficacy.

The GTPase domain (ROC-COR) of LRRK2 contains the residues from amino acids 1335–1878, accounting for ~7% of the full length protein. PD-linked mutations within the GTPase domain (eg. R144C/G) alter either GTP binding or GTPase activity [1–3,6,12]. Abolished GTP binding by the K1347A mutation attenuates LRRK2 kinase activity [6]. This leads to suppression of mutant-LRRK2-induced neuronal degeneration [6], and suggests that the GTPase domain is a tractable target for therapeutic intervention. In addition, the crystal structure of the LRRK2 GTPase domain is different from other small GTPases (eg, Ras, Rho) that could lead to development of potential inhibitors that only target LRRK2. Our recent studies have identified a GTP binding inhibitor, 68, that can reduce LRRK2 GTP binding activity but do not alter LRRK1 activity [13]. Moreover, 68 can reduce LRRK2 kinase activity and protect against mutant LRRK2 toxicity [13].

One of the challenges in developing therapeutics for neurodegenerative disorder is to improve both specific bioactive potency and blood-brain barrier penetration (BBB) simultaneously [11]. Many agents have failed to be developed into clinical drugs due to their low efficacy in brains [11]. Compound 68 is a potent inhibitor of LRRK2 GTP binding activity *in vitro*, with inhibitory activity in the low nanomolar range. However, 68 displays weak BBB permeability that limits the application of these inhibitors in animal models [13]. Herein we reported the design and synthesis of a novel analog of 68, compound FX2149, which not only kept the inhibition of LRRK2 GTP binding and kinase activities but also showed improved *in vivo* efficacy due to its enhanced BBB permeability. We further characterized the pharmacological effects of FX2149 using *in vitro* and *in vivo* PD models. Our studies provided a novel LRRK2 GTP binding inhibitor, FX2149, with a more efficient brain efficacy for future pathogenesis and therapeutic studies.

## Materials and Methods

### Materials, reagents, and animals

Anti-Flag antibodies were from Sigma (St. Louis, MO, USA). Anti-LRRK2 and anti-phospho-LRRK2 antibodies were from Michael J. Fox Foundation. Anti-isolectin B4, anti-4E-BP, anti-phospho-4E-BP and anti-tyrosine hydroxylase (TH) were from Cell Signaling Technology (Beverly, MA, USA). Compound 68 was custom ordered from Chembridge. LipofectAMINE Plus reagent and cell culture media were from Invitrogen (Carlsbad, CA). FX2149, FX2151, and 68 were dissolved in 0.1% DMSO/water solution for *in vitro* biochemistry and cell culture experiments. FX2149 and 68 were dissolved in 10% DMSO/0.9% saline for *in vivo* testing using mouse models. Wild type and G2019S-LRRK2-BAC transgenic mice [14,15] were ordered from Jackson Laboratory and maintained in the animal facility at University of Maryland School of Pharmacy, and the animal procedure protocol was approved by the Animal Use and Care Committee of University of Maryland.

### Synthesis of FX2149 [16, 25]

3-(Pyridine-3-sulfonamido)benzoic acid, 4 was synthesized as following steps. To a solution of ethyl 3-aminobenzoate methanesulfonate (**1**, 2.80 g, 11 mmol) in THF (30 mL) was added pyridine-3-sulfonyl chloride, **2** (1.77 g, 10 mmol), followed by triethylamine (2.1 mL, 15 mmol). The reaction mixture was allowed to stir at room temperature for 24 h and then concentrated. The crude product was purified with flash chromatography (EtOAc:hexanes, 1:4–1:1) to give ethyl 3-(pyridine-3-sulfonamido) benzoate, **3**, as a white solid (2.8 g, 9.2 mmol, 92%). The resulting compound **3** was dissolved in methanol (30 mL). To this solution was added NaON (1 N, 10 mL) drop wise. The reaction mixture was allowed to stir at 60°C for 16 hours and then cooled to room temperature. Methanol was removed by rotary evaporation, and the resulting bright yellow solution was acidified to pH 2 using HCl (4 N). Filtration under vacuum gave a white solid, which was further washed by HCl (1 N, 3 × 15 mL) to yield 3-(pyridine-3-sulfonamido)benzoic acid, **4**, as a white solid (2.45 g, 8.8 mmol, 88% for two steps): ^1^H NMR (400 MHz, DMSO-*d*_6_) δ 7.37–7.41 (m, 2H), 7.55–7.67 (m, 2H), 7.68 (s, 1H), 8.10–8.12 (d, *J* = 7.6 Hz, 1H), 8.77–8.78 (d, *J* = 4.0 Hz, 1H), 8.87–8.88 (d, *J* = 4.0 Hz, 1H), 10.20–11.20 (br s, 1H), 12.50–13.50 (br s, 1H); ^13^C NMR (100 MHz, DMSO-*d*_6_) δ 121.4, 124.9, 125.0, 125.9, 130.2, 132.3, 135.1, 136.0, 137.8, 147.4, 154.1, 167.0; LC-MS (M—H^+^) calcd for C_12_H_10_N_2_O_4_S 277, found 277.

*N*-Propyl-3-(pyridine-3-sulfonamido)benzamide (FX2149) was synthesized as following steps. To a mixture of carboxylic acid, **4** (556 mg, 2.0 mmol), EDC (575 mg, 3.0 mmol), and HOBt (460 mg, 3.0 mmol) was added *N*,*N*-dimethylformamide (DMF, 8.0 mL), followed by propylamine (200 *μ*L, 2.4 mmol). The reaction mixture was heated at 40°C for 24 hours. DMF was removed by rotary evaporation. To the resulting residue was added H_2_O (10 mL) to give a white slurry. Filtration under vacuum gave a white solid, which was further washed using H_2_O (4 × 10 mL). The product was further purified by recrystallization using CH_2_Cl_2_/hexanes give *N*-propyl-3-(pyridine-3-sulfonamido)benzamide (FX2149) as a white solid (515 mg, 1.61 mmol, 81%): ^1^H NMR (400 MHz, CDCl_3_) δ 0.94–0.98 (t, *J* = 7.2 Hz, 3H), 1.59–1.65 (m, 2H), 3.43–3.48 (q, *J* = 6.4 Hz, 2H), 6.37 (br s, 1H), 7.28–7.40 (m, 3H), 7.54–7.56 (d, *J* = 7.2 Hz, 1H), 7.75 (s, 1H), 8.09–8.11 (d, *J* = 8.0 Hz, 1H), 8.70 (s, 1H), 9.00 (s, 1H), 9.14 (s, 1H); ^13^C NMR (100 MHz, CDCl_3_) δ 11.4, 22.8, 42.0, 121.1, 122.5, 123.9, 124.0, 129.6, 135.5, 135.7, 137.4, 147.3, 152.4, 166.8; LC-MS (M + H^+^) calcd for C_15_H_18_N_3_O_3_S 320, found 320.

### Cell culture, LRRK2 constructs, and transfection

Human embryonic kidney HEK293T and human neuroblastoma SH-SY5Y cells were from ATCC (Manassas, VA, USA) and grown in the media as described previously [6,13]. The Flag tagged wild type, G2019S, R1441C, Y1699C, and G2019S-K1347A constructs were described previously [6]. Transient transfections were performed using Lipofectamine and PLUS Reagents (Invitrogen) according to the manufacturer's protocol.

### Immunoprecipitation (IP) and Western blot analysis

IP was performed using anti-FLAG-agarose (Sigma) as described previously [6,13]. For Western blot analysis, the resulting immunoprecipitates or cell lysates were run with 4–12% NuPAGE Bis-Tris gels and transferred onto polyvinylidene difluoride membranes (Invitrogen). The membranes were blocked with 5% nonfat milk and then incubated with various primary antibodies followed by secondary antibody detection as described previously [6,13]. Enhanced chemiluminescence (ECL) reagents were used to detect proteins on the membranes.

### LRRK2 GTP binding and phosphorylation (kinase) assays

GTP binding assays were performed using GTP-agarose beads (Sigma) as described previously [6,13]. Lysates of HEK 293T cells expressing LRRK2 proteins were incubated with 68 or FX2149 at various concentrations for 1h. The GTP-agarose beads were added for an additional 2 h. The samples were subjected to Western blot analysis using anti-Flag antibodies. LRRK2 kinase assays were performed using LRRK2 phosphorylation and *in vitro* ^32^P incorporation methods as described previously [6,13].

For LRRK2 phosphorylation assays, HEK293T cells were transfected with various LRRK2 variants for 36 h, then were incubated in media without serum for 12 hours. The cells were left untreated or treated with 68, or FX2149 for 1 h, and then were harvested with lysis buffer (Cell Signaling). The resulting cell lysates were immunoprecipitated using anti-Flag antibodies to pull down Flag-tagged LRRK2. The immunoprecipitates were subjected to Western blot using anti-phospho-LRRK2 antibodies (S2032 or S935) as described previously [13]. *In vitro* ^32^P incorporation was performed using purified LRRK2 that were left untreated or treated with 68 or FX2149 for 1 h. The samples were then incubated with the kinase reaction buffer containing 500 μM ATP and 10 μCi of [γ-^32^P]ATP (3,000 Ci/mmol) for 30 min. The LRRK2 autophosphorylation with ^32^P incorporation was separated by SDS/PAGE gel and quantified with a phosphoimager (Bio-Rad Molecular Imager).

### LRRK2 toxicity assays

SH-SY5Y cell viability assays were conducted as described [6,13]. Cells were co-transfected with GFP and various pcDNA3.1-LRRK2 plasmids at a 1:15 ratio for 24 h in 10% FBS OPTI-I media and then changed to DMEM with N2 supplement for 24 h. Compounds were added after 4-h transfection. Cell viability was measured by counting the healthy viable cells that contained at least one smooth extension (neurite) that was twice the length of the cell body from 20 randomly selected fields using fluorescence microscopy [6,13]. TUNEL assays were performed according to the manufacturer's instructions as described previously [6,13]. The experiments were repeated three times in duplicate. The quantification for LRRK2 toxicity was performed by an investigator who was blind to transfection groups.

### LPS-based preinflammatory mouse model and immunohistochemical analysis

G2019S-LRRK2-BAC transgenic mice were anesthetized with isoflurane and injected with LPS (15,000 endotoxin units, 5 μg, Sigma) for each mouse in the substantia nigra pars compacta (SN) unilaterally as described previously [13,16]. The brain coordinates for injection of LPS were -1.1 mediolateral (ML), -3.4 anteroposterior (AP), and -3.9 dorsoventral (DV) related to bregma. FX2149 and 68 were injected i.p. 1hour prior to LPS injection at doses of 0, 10, or 20mg/kg. FX2149 and 68 were then injected (i.p.) twice daily for three days. Brain tissues were harvested with 4% paraformaldehyde (PFA) perfusion. The frozen brain sections through the SN at 30 μm were subjected to immmunohistochemical analysis as described previously [13]. Brain sections were incubated with various primary antibodies including anti-isolectin B4, anti-phosphorylated LRRK2 S935, anti-LRRK2, and anti-TH (Milipore) antibodies. Then the sections were incubated with fluorescent secondary antibodies including Alexa Fluor 568 goat anti-mouse (rabbit) IgG (Invitrogen) and Alexa Fluor 488 goat anti-mouse (rabbit) IgG. Some sections were added with anti-rabbit (mouse) biotinylated secondary antibody and avidin–biotin–peroxidase complex (Vector Laboratories), and detected by diaminobenzidine (DAB, Sigma). The images of brain sections were taken using a Zeiss Axioskop 2 microscope and a Zeiss Axiocam camera, and processed using Adobe Photoshop (VII) software. The continuing middle brain section series from each mouse brain were sampled by 6 section intervals for fluorescent density quantification of the SN areas. The quantification of fluorescence density was performed by unbiased stereology with an investigator who was blind to experiment groups.

### Data Analysis

Quantitative data were shown as arithmetic means ± SEM from three separate experiments. Statistically significant differences among groups were analyzed by ANOVA using Sigmastart 3.1 statistical software (Aspire Software International, VA). A *p* value <0.05 was considered significant.

## Results

### Design and synthesis of FX2149

**Fig 1:**
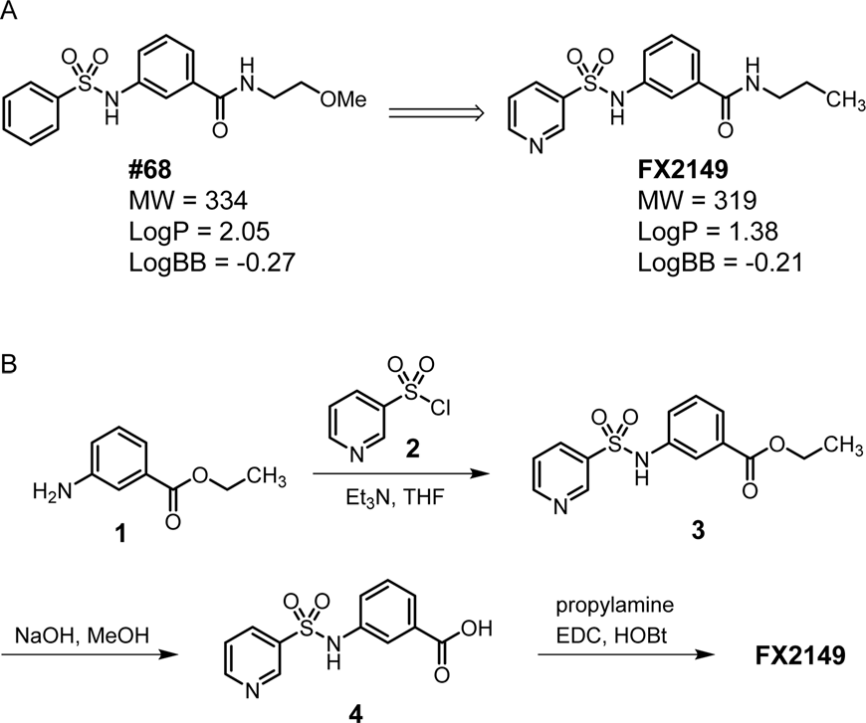
A. Chemical structures of 68 and FX2149; B. Synthesis of FX2149.

Given that 68 potently inhibits LRRK2 GTP binding and kinase activity *in vitro* [13], we kept its scaffold structure to retain the inhibition of GTP binding and kinase activity. A compound with good BBB permeability requires a Log P value between 1.0 and 3.0 and a Log BB value between -2.0 and 1.0 [17]. To optimize the BBB permeability of 68, we used a pyridine-3-sulfonamide group to replace the phenyl-sulfonamide head of 68 (Fig. 1A). Weakly basic groups, such as the pyridinyl group, are commonly present in therapeutic agents targeting the central nervous system [18]. Moreover, the 2-methoxy-ethyl tail of 68 was substituted by a propyl group to reduce the number of H-bond acceptors to fit the binding site of the LRRK2 GTPase domain. Compound FX2149 was calculated to have increased hydrophilicity (LogP = 1.38 vs 2.05 for 68) and enhanced BBB permeability (LogBB = -0.21 vs -0.27 for 68, calculated by using ACD/Labs Suite 5.0).

The synthesis of compound FX2149 involved a three-step procedure as shown in Fig. 1B. First, ethyl 3-aminobenzoate methanesulfonate, **1**, was treated with pyridine-3-sulfonyl chloride, **2**, in the presence of triethylamine (Et_3_N) at room temperature to generate compound **3** as a mixture of rotamers in excellent yields [19]. Next, saponification of ethylester in compound **3** using aqueous NaOH yielded carboxylic acid, **4**, in high yields. Finally, compound **4** was coupled with propylamine using *N*-ethyl-*N*′-(3-dimethylaminopropyl) carbodiimide hydrochloride (EDC) and 1-hydroxybenzotriazole hydrate (HOBt) to provide FX2149 in high yields [25]. Compound FX2149 was purified by flash chromatography with over 95% purity as described in the method section.

### FX2149 reduced LRRK2 binding with GTP

**Fig 2:**
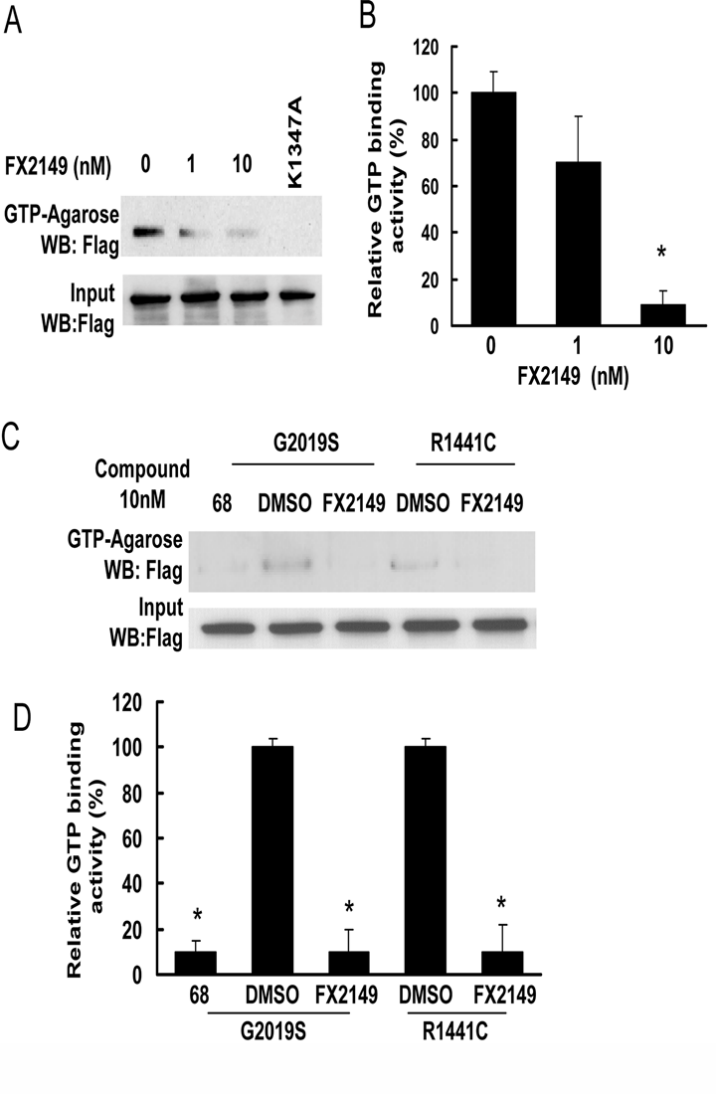
FX2149 inhibits LRRK2 GTP binding activity. WT or mutant LRRK2 was pulled down from lysates of transfected HEK293T cells using GTP-agarose in the absence or presence of FX2149 at 1 and 10 nM concentrations. The resulting precipitates were subjected to western blot analysis using anti-Flag antibodies. A and C. Representative blots from GTP binding assays. B and D. Quantification of A and C. K1347A-LRRK2, non GTP binding genetic control. All experiments were repeated three times with similar results. *p<0.05 by ANOVA, *vs* vehicle control.

To assess the effects of FX2149 on LRRK2 GTP binding activity, a GTP binding assay was employed using GTP-agarose as described previously [13]. GTP-agarose pulled down LRRK2 from the lysates of HEK293T cells expressing human LRRK2. Incubation of FX2149 with GTP-agarose significantly reduced LRRK2 binding with GTP (Fig. 2A and 2B). FX2149 at 10 nM concentration reduced LRRK2 GTP-binding activity by ~90% (Fig. 2A). Similar to the effects of 68 [13], FX2149 reduced the PD-linked mutant LRRK2 variants (G2019S and R1441C) that bound with GTP (Fig. 2C and 2D).

### FX2149 reduced LRRK2 kinase activity

**Fig 3:**
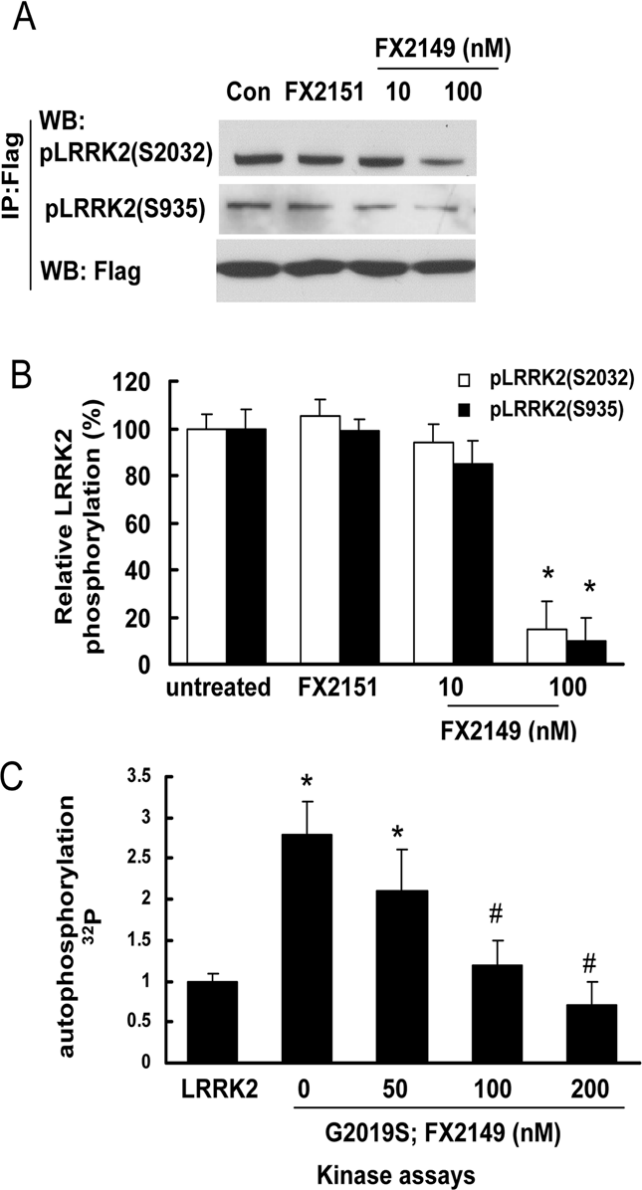
FX2149 reduced LRRK2 phosphorylation. A and B. HEK293T cells were transiently transfected with Flag tagged G2019S-LRRK2 construct for 36 h and then starved with no serum media for 12 hours. Then vehicle, FX2151 (10 μM, a non-effective analog of 68), or FX2149 (10 and 100 nM) were treated for 1h. Cell lysates were subjected to immunoprecipitation using anti-Flag antibody followed by Western blot analysis using anti-phospho-LRRK2 (S2032 or S935) antibodies. A. Representative blots from three repeated LRRK2 phosphorylation assays. B. Quantification of LRRK2 phosphorylation from A. *p< 0.05 by ANOVA compared with FX2151 treated group. C. LRRK2 and G2019S-LRRK2 were purified from cell lysates using anti-LRRK2 immunoprecipitation. The purified LRRK2 variants were incubated with FX2149 (0, 50, 100, or 200 nM) for 1 h and then subjected to *in vitro* kinase assays using γ-^32^p -ATP incorporation method. LRRK2 autophosphorylation was quantified from three repeated experiments. *p< 0.05 by ANOVA compared to wild type LRRK2. ^#^ *p* < 0.05 by ANOVA compared to G2019S-LRRK2 treated with vehicle.

To further assess whether FX2149 alters LRRK2 phosphorylation (kinase activity), HEK293T cells expressing mutant G2019S-LRRK2 were treated with FX2149 at concentrations of 0, 10, and 100 nM for 1 h. Cell lysates were subject to LRRK2 autophosphorylation (kinase activity) assays. FX2149 at 100 nM concentration significantly reduced G2019S-LRRK2 phosphorylation at residues S935 and S2032 by ~90% (Fig. 3A and 3B). We further validated these results by *in vitro* kinase assays showing the similar inhibition of G2019S-LRRK2 kinase activity by FX2149 (Fig. 3C). These findings indicated that FX2149 (100 nM) reduced LRRK2 kinase activity similar to that of 68 at 10 nM concentration [13]. An inactive analog of 68, FX2151, did not alter the LRRK2 phosphorylation at 10 μM concentration, which is consistent with our previous findings [13].

### FX2149 attenuated mutant LRRK2-induced toxicity in SH-SY5Y cells

**Fig 4:**
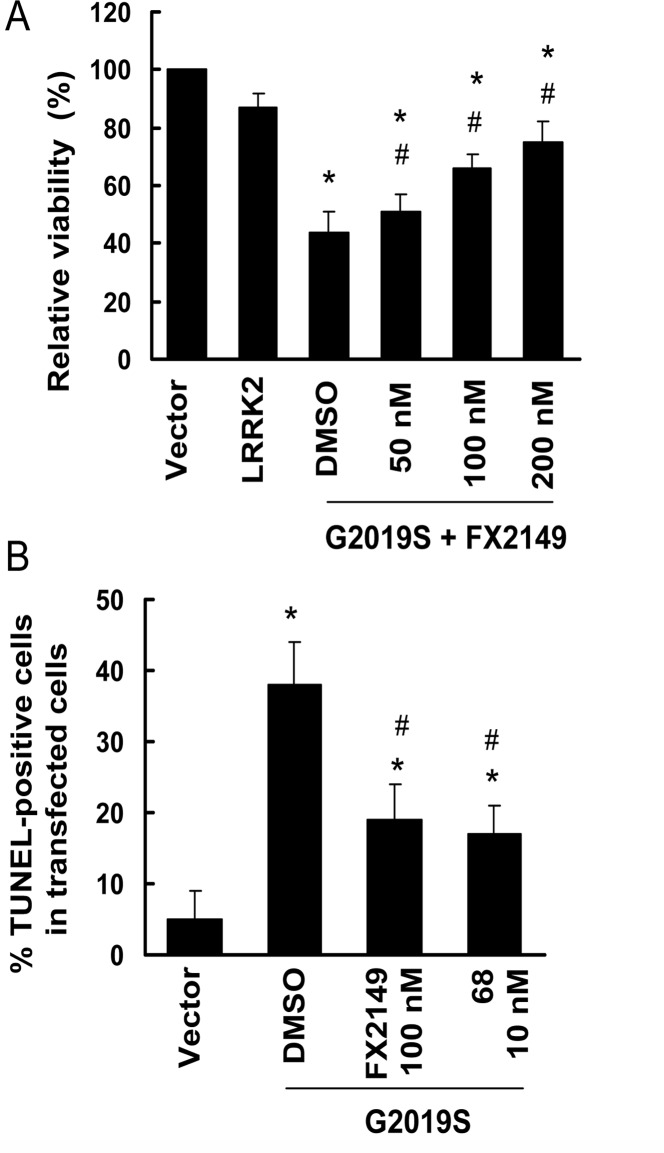
FX2149 attenuated G2019S-LRRK2-induced neuronal degeneration in SH-SY5Y cells. SH-SY5Y cells were co-transfected with GFP and various pcDNA3.1-LRRK2 plasmids at a 1:15 ratio as described in the method section. After 4-h transfection, cells were treated with FX2149 for 48 hours. A. Cell viability was measured by counting the healthy viable GFP positive cells that contained at least one smooth extension (neurite) that was twice the length of the cell body. *p< 0.05 by ANOVA compared to wild type LRRK2. ^#^ *p* < 0.05 by ANOVA compared to G2019S-LRRK2 treated with vehicle. B. TUNEL assays. The experiments were repeated three times. *p < 0.05 by ANOVA compared to vector control. ^#^ *p* < 0.05 by ANOVA compared to G2019S-LRRK2 treated with vehicle.

GTP binding activity and elevated kinase activities have been implicated in PD-linked mutant, G2019S-LRRK2, resulting in neurodegeneration [6,13]. SH-SY5Y cells contain dopamine and are often used as a PD cell model [6]. To assess whether FX2149 alters mutant LRRK2-induced neuronal degeneration, G2019S-LRRK2 construct transiently transfected into SH-SY5Y cells was used as a toxicity model as previously described [6]. Treatment of FX2149 significantly increased the viability of cells expressing G2019S-LRRK2 compared with vehicle treated cells (Fig. 4A). Moreover, FX2149 at 100 nM significantly reduced the TUNEL-positive cells expressing mutant G2019S-LRRK2 and had effects equivalent to that of 68 at 10 nM (Fig. 4B).

### FX2149 was more efficient in reducing LRRK2 GTP binding and kinase activities in transgenic mice brains than 68

**Fig 5:**
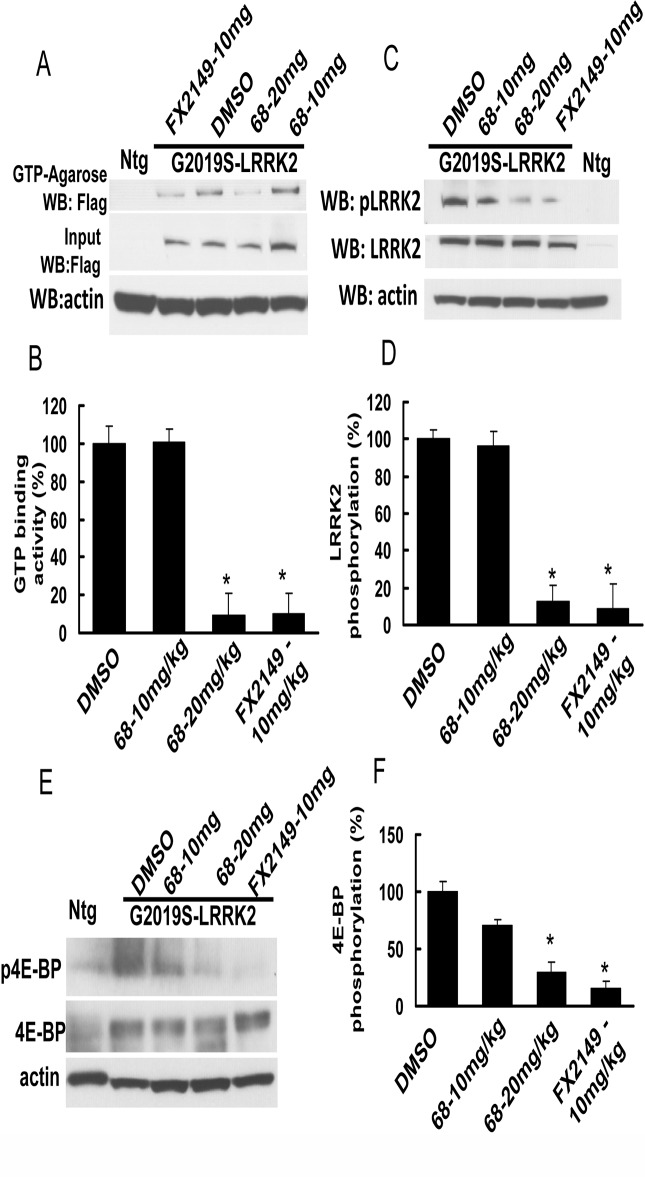
FX2149 improved the brain penetration and inhibition of LRRK2 GTP binding and kinase activities. FX2149 (10 mg/kg) and 68 (10 and 20 mg/kg) were injected intraperitoneally into G2019S-LRRK2 BAC transgenic mice at 6–12 weeks of age for 1 hour. There were 6 mice in each experimental group. The brain homogenates were used to detect LRRK2 GTP-binding and kinase activities. A and B, LRRK2 GTP-binding assays. C and D, LRRK2 phosphorylation assays using anti-phospho-LRRK2 antibodies. E and F, FX2149 reduced G2019S-LRRK2-induced 4E-BP phosphorylation determined by anti-phospho-4E-BP western blot analysis. Ntg: non-transgenic mouse. *p < 0.05 by ANOVA compared with G2019S-LRRK2 transgenic mice treated with vehicle.

To assess the effects of FX2149 on LRRK2 in brains compared with 68, both 68 and FX2149 were injected intraperitoneally into G2019S-BAC-LRRK2 transgenic mice at 10 and 20 mg/kg doses. One hour after the injection, the mouse brain homogenates were subjected to LRRK2 GTP binding and kinase assays. Both 68 (20 mg/kg) and FX2149 (10 mg/kg) reduced LRRK2 GTP binding activity in mouse brains (Fig. 5A and 5B). While FX2149 at a 10mg/kg dose had the equivalent GTP binding inhibition as 68 at a 20 mg/kg dose, compound 68 at 10 mg/kg dose did not alter brain GTP binding activity 1 hour after injection. Moreover, both 68 (20 mg/kg) and FX2149 (10 mg/kg) also significantly reduced brain LRRK2 kinase activity (Fig. 5C and 5D). To further confirm the effect of FX2149, we also assessed a LRRK2 downstream effector, 4E-BP phosphorylation. 4E-BP is a transcription factor that can be phosphorylated by LRRK2 [20]. We found that both 68 and FX2149 reduced 4E-BP phosphorylation in mouse brains (Fig. 5E and 5F). FX2149 at a 10mg/kg dose reduced 4E-BP up to 15% of the untreated control group, while 68 at a 20 mg/kg dose reduced 4E-BP up to 15% of the untreated control group. These data indicated that FX2149 was taken up into brains at a greater extent and had more potent efficacy in inhibiting GTP binding and kinase activity in mouse brains when compared with 68.

### FX2149 reduced LPS-induced microglia activation and LRRK2 upregulation in mice

**Fig 6:**
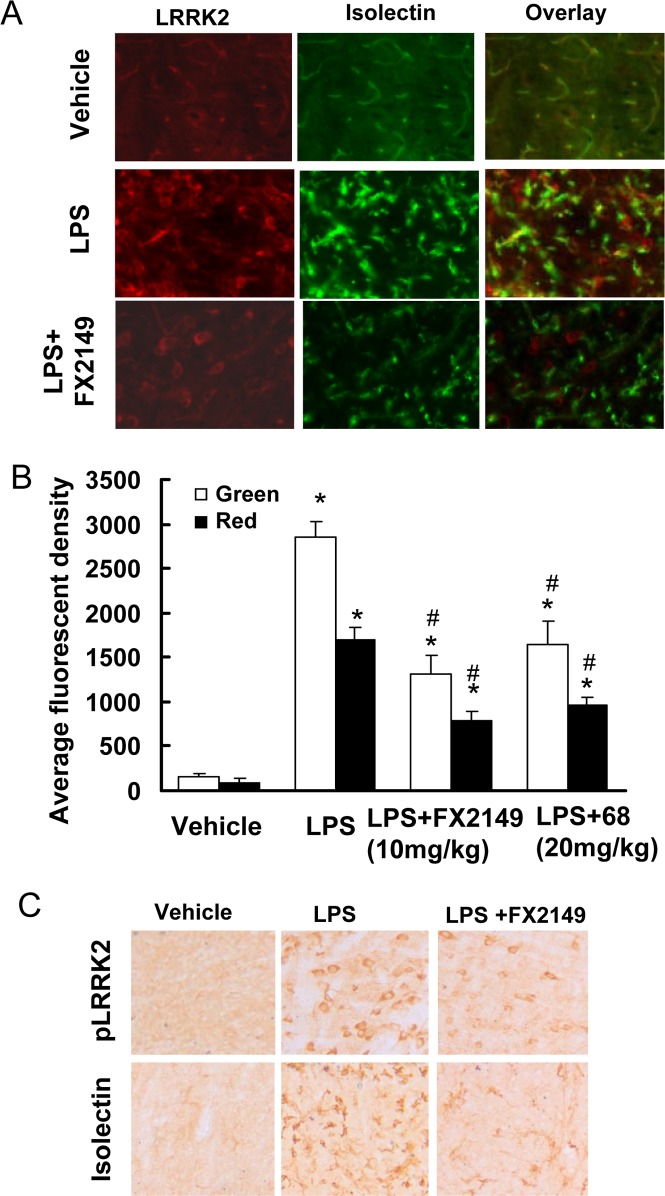
FX2149 reduced LPS-induced microglia activation and LRRK2-upregulation. G2019S-LRRK2 BAC transgenic mice (6–12 weeks) were injected with LPS (5 μg) and FX2149 (10 mg/kg) as described in the methods section. Serial coronal sections through the substantia nigra were subjected to immunohistochemistry analysis. A. Representative immunofluorescent images with anti-isolectin (green) and anti-LRRK2 (red) staining. B. Quantification of immunofluorescence of A by unbiased stereology. *p < 0.05 by ANOVA compared with vehicle group. ^#^ *p* < 0.05 by ANOVA compared with LPS treated group. C. Representative immunostaining with anti-phospho-LRRK2-S935 and anti-isolectin B4 (marker for microglia) antibodies by DAB detection.

To further characterize the pharmacological effects of FX2149 in brains, a LPS-based mouse neuroinflammation model was used as described previously [13,16]. Consistent with previous findings [13,16], injection of LPS resulted in significant increases in LRRK2 expression, phosphorylation, and microglial activation in the substantia nigra compared with vehicle controls (Fig. 6). Treatment of mice with FX2149 significantly reduced LPS-induced LRRK2-positive immunostaining compared with vehicle controls, but it did not alter LRRK2 cytoplasmic localization. Moreover, the anti-phosphoryated-LRRK2 immunoactivity was also significantly reduced in the FX2149 treated group. As in our previous study of 68 at 20 mg/kg [13], FX 2149 at a 10 mg/kg dose significantly reduced LPS-induced isolectin B4 (microglia marker) positive immunostaining in the substantia nigra. There was a ~43% isolectin B4 immunoactivity in the FX2149 treated group compared with the LPS alone treated group. In comparison, the immunoactivity of isolectin B4 in the 68 treated group (20 mg/kg) was ~56% (Fig. 6B). Consistent with previous findings [13], anti-TH (dopaminergic neuron marker) immunostaining in the subtantia nigra did not change among various treatment groups and the control group, indicating that LPS treatment did not alter dopaminergic neuron degeneration in this acute inflammation condition.

## Discussion

In previous studies, we identified and characterized a LRRK2 GTP binding inhibitor, 68 [13]. To improve *in vivo* effects of 68, we designed and synthesized a novel analog of 68, compound FX2149, to improve brain permeability. The *in vitro* biological characterization of FX2149 demonstrated that it inhibited LRRK2 GTP binding and kinase activity, and protected against mutant LRRK2 toxicity at 10–100 nM concentrations, which is a bit lower in efficacy than 68. However, FX2149 showed increased *in vivo* efficacy, with a more than 2-fold improvement over 68. FX2149 significantly reduced LPS-induced microglia activation and LRRK2 upregulation at a significantly lower dose than 68. These findings demonstrated that FX2149 is a better lead GTP binding inhibitor with improved brain penetration for future drug development and pathogenesis studies.

The major pathology area of PD is in the substantia nigra [1,2]. Consequently, for therapeutic agents to prevent neurodegeneration, they must cross the BBB [11,17]. Most neuroprotective compounds fail further development due to either a lack of high potency in brains or poor BBB permeability [11,17]. Based on the chemical scaffold of 68, a new analog, FX2149, has been synthesized by substituting the phenylsulfonyl fragment of 68 with a pyridine-3-sulfonyl group, while replacing the methoxyethyl tail of 68 with a propyl tail [19,21]. FX2149 has similar effects in inhibition of LRRK2 GTP binding activity compared with 68, although it has less potency than 68 in *in vitro* assays. FX2149 at 100 nM inhibited LRRK2 kinase activity equivalent to 68 at 10 nM by *in vitro* phosphorylation assays. However, FX2149 had a potent efficacy in inhibiting LRRK2 GTP binding and kinase activities by *in vivo* testing with the LRRK2 transgenic mouse model. FX2149 at 10 mg/kg had an approximately equivalent GTP binding and kinase inhibition effect as seen in 68 at 20 mg/kg. FX2149 at 10 mg/kg had a stronger effect in reducing mutant G2019S-induced 4E-BP phosphorylation compared with the treatment group of 68 at 20 mg/kg. 4E-BP is a stress-related transcription factor and increases in phosphorylation is believed to contribute to neuronal degeneration [20]. Taken together, these results demonstrated that FX2149 had better brain penetration efficacy for animal studies as required for PD intervention.

The loss of dopaminergic (DA) neurons in the substantia nigra is an early and key pathological hallmark of PD [21–23]. Disruption of LRRK2 GTP binding by genetic mutation reduces LRRK2 kinase activity, thereby suppressing neuronal degeneration [6,13]. Similar to the effects of 68 [13], our results showed that compound FX2149 reduced LRRK2 GTP binding and kinase activities, and significantly attenuated mutant LRRK2-induced neuron degeneration in *in vitro* cell culture models. Another feature of neurodegeneration is microglia activation and inflammation in brains [24]. Microglia activation releases various inflammatory cytokines which trigger or facilitate dopaminergic neuronal loss in PD [24]. Recent studies show that the preinflammatory agent, LPS, elevates LRRK2 expression and phosphorylation in activated microglia in mice [13,16,25]. Our results showed that FX2149 at 10 mg/kg significantly reduced LPS-induced microglia activation by 57% compared with the vehicle treated LPS mice group. In comparison, 68 at 20 mg/kg only reduced LPS-induced microglia by 44% (Fig. 5B). These results further validated the significantly improved *in vivo* effects of FX2149 compared to 68 in the LPS-based animal model.

In summary, these studies provided a novel GTP binding inhibitor, FX2149 (analog of 68), and further proved that GTP binding regulates LRRK2 kinase activity. Disruption of GTP binding activity may be an effective strategy to prevent neuron degeneration for PD and other LRRK2-related disorders.
